# Commendable

**Published:** 1887-03

**Authors:** 


### COMMENDABLE.

Doctor Gann, of Bolton, attends promptly every meeting of the Atlanta Society of Medicine. When one takes into consideration that the Doctor's home is seven miles from Atlanta; that the Society meets once a week, and that the hour is 7½ p. m., one must admire, not only the pluck and assiduity of the young practitioner but to indulge in some criticism upon the apathy, indifference and quasi-hostility of some professional brethren who cannot afford to spend an hour with their colleagues, and perhaps illustrate thereby the fact that true progressiveness is the offspring of collaboration.

